# From awareness to agency: a Freirean analysis of critical consciousness and social justice among international medical students in the UK

**DOI:** 10.3389/fmed.2026.1854824

**Published:** 2026-06-10

**Authors:** Shafeena Anas, Jenan Younis, Maria Tsouroufli

**Affiliations:** Brunel Medical School, Brunel University London, Uxbridge, United Kingdom

**Keywords:** critical consciousness, critical pedagogy, international medical students, medical education, social justice

## Abstract

Social justice is increasingly emphasised in medical education, yet students often struggle to translate awareness of inequity into meaningful action. Drawing on Paulo Freire’s critical pedagogy, this study explores how international medical students conceptualise social justice, encounter inequity within clinical and educational environments, and perceive their capacity to act as agents of change. Given the growing internationalisation of UK medical schools, understanding how international learners develop critical consciousness and experience inequity is essential.

**Methods:**

A qualitative study was conducted using three online focus group studies with 11 Year 2 international medical students at a UK medical school. Data was audio-recorded, transcribed verbatim and thematically analysed using a Freirean framework. Analysis focused on students’ expression of critical consciousness, experiences of structural inequity, engagement with the hidden curriculum, sense of belonging and perception of agency and praxis.

**Results:**

Four interconnected themes were identified. (1) Emerging critical consciousness: students demonstrated early awareness of social injustice, recognising how poverty, racism, disability, immigration status and biased curricular representations shaped health outcomes. (2) Witnessing inequities in clinical practice: clinical placements in General Practice (GP) settings exposed students to structural constraints such as language barriers, limited resources, inaccessible services and challenges faced by asylum seekers, reinforcing their understanding of health inequity as systemic rather than individual. (3) The hidden curriculum, inequity and belonging: while students identified financial and representational inequities within medical education, they also described a strong sense of belonging fostered through stable Team-Based Learning (TBL) groups, supportive peers and approachable staff. For international students this relational inclusion provided psychological safety and enabled engagement with justice-focused reflection. (4) Awareness without agency: despite growing critical consciousness, students reported limited power to enact change, citing hierarchical norms, lack of authority and resource-constrained systems, reflecting a predominantly banking model of education.

**Conclusion:**

International medical students demonstrated emerging critical consciousness and strong relational and institutional belonging, yet faced systemic barriers that constrained their ability to translate awareness into action. Medical education must move beyond awareness-raising to intentionally create opportunities for praxis, address the hidden curriculum, and support faculty to foster action-oriented, socially just learning environments. Integrating belonging with structured opportunities for participation and change is essential to developing critically conscious, socially responsive future practitioners.

## 1 Introduction

Social justice is increasingly recognised as a core imperative within Health Professions Education (HPE) (1). Social justice has been described as the ‘heart of medical education’ (2) with an emphasis on fair distribution of rights, opportunities, and resources within society and the reduction of health disparities (2, 3). Kumagai and Lypson (4) define social justice as “the open acknowledgement of the dignity and autonomy of, and delivery of high-quality medical care to, all members of society, regardless of gender, race, ethnicity, religion, sexual orientation, language, geographic origin, or socioeconomic background.” van Schalkwyk et al. (1) describes social justice as a complex construct, linking to concepts of equity (5), health equity (47), social accountability (2) and cultural diversity, competence and humility (4, 6, 7).

Across HPE, this translates predominantly into curricular efforts aimed at equipping students with the knowledge of social determinants of health, promoting health equity and fostering advocacy (8, 47). Hixon et al. (2) emphasise the role of physicians in promoting equitable healthcare access. Similarly, Khan et al. (9) highlight that medical students are expected to develop competencies in recognising and intervening in the social and structural determinants that shape patient health outcomes. Van Schalkwyk et al. (1) explain that faculty development for teaching social justice requires “questioning the causes of health inequity and intervening in educational HPE contexts and systems with a view to transforming them into more socially just spaces” (47). Medical curricula are also expected to incorporate an understanding of the factors contributing to social determinants of health, including socioeconomic status, education, housing, and access to care (8, 9). Issues of representation within medical curricula further reinforce inequities, with underrepresentation of minoritised groups in teaching materials and research linked to diagnostic inaccuracies and disparities in patient care (5, 10, 11). Students are expected to recognise how these factors shape patient experiences and contribute to differential outcomes (9). This aligns with broader educational goals of fostering socially responsive practitioners who can engage with the complexities of patient care (8).

Within this social justice orientation, students are frequently positioned as future agents of change, with an ethical responsibility to address health inequities and advocate for vulnerable populations (12, 13). However, structural barriers such as limited resources, time pressures, and organisational constraints can limit opportunities for action (14). For students, exposure to these constraints during clinical placements can create a tension between theoretical knowledge and practical reality. Medical education is characterised by deeply entrenched hierarchical structures, in which students occupy positions of relatively low power (15, 16). It is argued that while medical education increasingly promotes awareness of social justice issues, it often fails to provide opportunities for students to engage in meaningful action. This gap between awareness and action is central to understanding the limitations of current approaches to social justice education (14, 17).

At the same time, distributive models of justice have been critiqued for insufficiently addressing the relational, cultural, and epistemic dimensions through which inequity is reproduced in medical education itself (5, 18). As Brown et al. (18) argue, the concept of social justice remains insufficiently defined in medical education, often defaulting to traditional understandings of equality without interrogating deeper structural and relational dynamics. Scholars argue that justice in HPE must move beyond addressing health inequities to interrogate power, recognition, representation, and the politics of knowledge (5, 11, 19, 20). Hidden curriculum practices, underrepresentation of minoritised bodies in teaching materials, the persistence of microaggressions, differential attainment and exclusionary institutional cultures demonstrate how medical schools can inadvertently perpetuate inequality even while even as they espouse commitments to equity (21–24). These structural inequities mirror broader societal hierarchies and highlight the limitations of surface-level EDI initiatives that fail to address underlying power dynamics (25). As Jain and Scott (26) argue, without intentional redesign of educational structures, medical schools risk perpetuating the very inequities they seek to dismantle. These insights suggest that social justice in medical education requires more than teaching about health inequity: it demands critical examination of how educational environments themselves enact injustice (18). Recent literature in medical education has increasingly called for a shift from distributive models of social justice, which focus on the allocation of resources, toward transformative approaches that address the underlying structures producing inequity (4, 14, 27). Transformative justice emphasises critical engagement with power, structural change, and the development of agency among learners, positioning medical education as a site not only of knowledge transmission but also of social change (4, 14).

Freire’s critical pedagogy offers a powerful conceptual framework for understanding social justice and the relationship between awareness, power, and action in medical education (4, 8, 17). Central to this framework is the critique of the ‘banking model’ of education, in which learners are positioned as passive recipients of knowledge without opportunities to interrogate or transform their social context (28). Within this banking model, students may acquire knowledge about social justice and health inequities but may not be equipped to challenge or transform the systems that produce them. This aligns with critiques of medical education that highlight its emphasis on knowledge acquisition over critical engagement and action (46).

Freire (28) advocates for the development of critical consciousness (conscientisation) - an awareness of social, political, and structural injustices and crucially, the capacity to act upon this awareness through praxis, defined as the integration of reflection and action (15, 29, 46). Many scholars have linked the concept of critical consciousness to social justice in medical education (1, 4, 8, 17). Kumagai and Lypson (4) define critical consciousness in medical education as ‘awareness of power and privilege and inequities that are embedded in social relationships’. van Schalkwyk et al. (1) state that critical consciousness in medical education is the ‘process of learning to perceive social political and economic conditions and taking actions against oppressive elements.’

Ross (8) proposes that critical pedagogy can inform medical education to (1) embed the wider social context of health in the curriculum, (2) prepare students for the complexities of the populations they will serve, (3) help them consider the effect of place (power and positionality) and (4) enable students to enact change which will help achieve equity. However, scholars have argued that while curricula teach about health equity and social determinants of health, opportunities for students to enact change remain limited by hierarchical structures, hidden curricula, and institutional constraints (30, 46). This raises important questions about how medical students understand social justice, how they encounter inequity within educational environments and how they perceive their developing agency.

These issues take on a particular significance within the context of international students in UK medical schools. International medical students in the UK are growing but remain under-researched as a group. International students navigate medical education and training from diverse cultural and sociopolitical backgrounds yet must make sense of justice issues within UK educational and healthcare structures marked by long-standing inequities. They also encounter additional barriers such as migration status, financial pressures and unfamiliar academic norms. Despite rapid internationalisation of UK medical schools, little is known about how international learners conceptualise social justice or how their positionalities shape their perception of power, marginalisation and agency.

This study addresses this gap by examining how international students understand social justice, how they experience inequity within educational and clinical contexts and how they perceive their capacity to function as agents of change. Using a Freirean analytical framework, it explores the tension between awareness and agency, and between institutional commitments to justice and the realities of medical education and training. In doing so, it seeks to contribute to ongoing efforts to move beyond distributive accounts of justice towards more relational, epistemic, and transformative approaches in medical education. The overall aim of the study was to explore EDI in medical education including the curriculum including the experience of medical students in the learning environment and the clinical environment.

## 2 Methodology

Embedded within a doctoral thesis, this research was conducted at a UK based medical school with a diverse international cohort. The study was conducted through focus groups and semi-structured interviews. The data drawn for this paper are from the focused group study with medical students. At the time the focus groups were conducted, the student participants were in their second year of study. The student participants in the study are all international students from diverse backgrounds.

### 2.1 Theoretical framework

The study adopted a qualitative, interpretive design informed by Paulo Freire’s critical pedagogy, which conceptualises education as political and relational practice shaped by power, inequality and the potential for transformation. Freire’s framework positions learners as active co-constructors of understanding and knowledge and not as passive recipients of knowledge. This theoretical positioning is relevant to examine how medical students make sense of social justice, health inequalities and their own emerging agency within medical education.

Guided by Freire’s principles, the study assumes that participants’ narratives are shaped by their experiences within educational structures that may reproduce or challenge inequity. The participants in this study were all international students in the UK and this methodology allowed us to centre students’ voices, acknowledge the sociopolitical conditions shaping their experiences, and seeks to highlight the tensions between awareness, agency and structural limitations.

**Figure 1:**
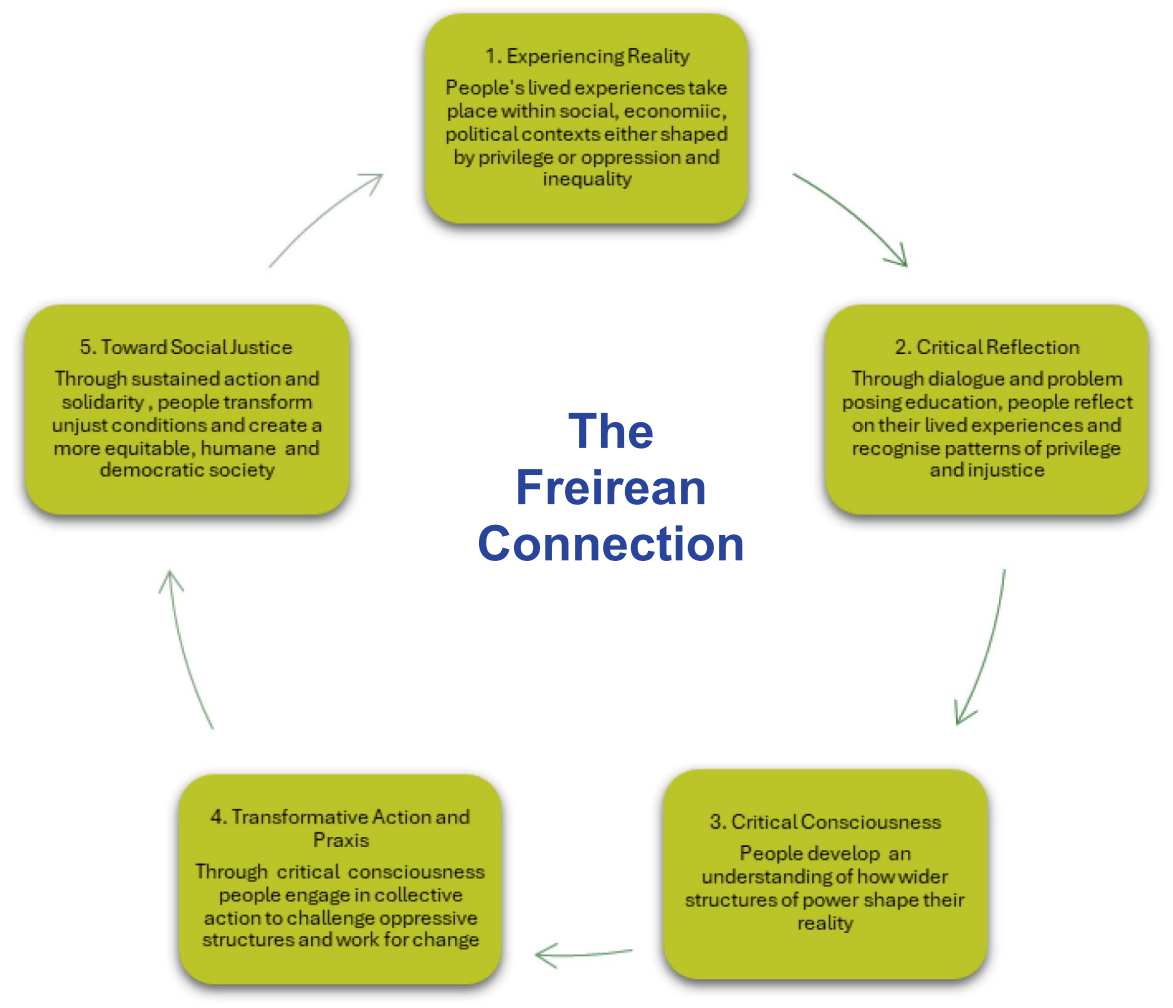
The Freirean connection: the critical consciousness pathway to social justice.

Figure 1 illustrates how Freire set out the connection between critical consciousness and social justice through critical pedagogy education.

### 2.2 Settings and participants

The study was conducted in a UK medical school offering a 5-year undergraduate MBBS programme. Current yearly intake is approximately 150 students with a total enrolled students of approximately 490 students with international students making up 60% of the cohort.

### 2.3 Data collection

Focus groups were selected as the method of data collection to align with Freirean principles of dialogue and collective sense-making (31). Focus groups enabled participants to co-construct meaning through interaction, reflection and questioning shared experiences, moving away from privileging individual narratives. Focus groups facilitated dialogic engagement, enabling participants to collectively interrogate experiences of inequity, consistent with Freirean problem-posing education. Given the study’s focus on social justice, power and belonging, focus groups provided a space for participants to collectively interrogate experiences of inequity, challenge assumptions and build on one another’s insights in ways that individual interviews may not facilitate.

The focus groups were conducted by an independent facilitator external to the medical school in order to address issues of power and hierarchy between student participants and facilitator. A semi-structured topic guide was used by the facilitator flexibly in order to facilitate dialogue through the use of open-ended prompts and follow up questions. Participants were encouraged to respond to, question and elaborate on one another’s contributions and the facilitator was able to adopt a non-hierarchical role, allowing discussions to evolve in response to participant discussions. While the core topics included questions such as “What you understand by the term social justice, particularly in medical education?, ‘Do you think different groups of medical students have different experiences in medical schools?’ participants had the opportunity to shape the direction and depth of discussions for example by talking about inequities they observed in GP settings when introduced to the topic of social justice. While belonging was another explicit topic, reflections and discussions on belonging emerged independently and led the facilitator to ask “It’s a really good point, because you’re talking about that feeling of belonging…How do you feel? Do you feel like you belong to the medical school community?”

A total of 11 Year 2 medical students took part in the focus groups. Three focus groups were conducted online via MS Teams and were audio-recorded and transcribed verbatim. Each focus group lasted between 75 and 90 min. Focus group 1 had four participants, focus group 2 had three participants and focus group 3 had four participants. Participants self-identified their gender and there were six male, four female, and one non-binary student. Second year medical students have early exposure to clinical experience with clinical placements in General Practice every 2 weeks from Year 1 of the programme.

### 2.4 Reflexivity and positionality

This study is situated within a reflexive qualitative paradigm recognising that knowledge is socially produced and shaped through positionalities, relationships and interpretive commitments of the research team. In line with reflexive thematic analysis (32), reflexivity was treated an as analytic practice acknowledging the researchers’ active role in meaning-making rather than claiming neutrality or a procedural statement of identity. The research team included three racially minoritised women: a migrant Professor specializing in Gender Equality and Critical Education, a Senior Academic in a medical school who is also a doctoral student specializing in EDI in medical education, critical education and a Medical Doctor who is also an educator in a medical school. The research team possessed diverse ethnic and national identities and expertise across multiple disciplines (gender studies, critical education, public health and medicine) and their commitment to social justice and critical pedagogy introduced sensitivity to issues of hierarchy, oppression and praxis. The research team’s intersecting identities shaped both the epistemological orientation of the study and analytic focus on power, marginalisation and agency within medical education. To ensure that interpretations were not overly deterministic, the team was attentive to reflexivity to recognise complexity in participants’ accounts.

Power dynamics were a central reflexive consideration throughout the study, particularly given the first author’s senior role within the institution where participants were enrolled. To address the risk of reinforcing academic hierarchy and shaping what the participants felt able to articulate, the focus groups with students were facilitated by a colleague external to the medical school. However, acknowledging Crabtree and Millers (33) framing of reflexivity, power cannot be fully mitigated through methodological procedures and is inherently featured in the research process. Therefore, participants’ articulation of belonging and restraint were treated as analytically meaningful reflecting their navigation within institutional power rather than simple expressions of satisfaction or comfort.

The team’s diverse disciplinary backgrounds shaped interpretation of the data with both convergence and divergence. The medical perspective provided a realistic insight into the constraints of clinical practice and professional socialisation processes, the critical education and sociological perspectives drew attention to the epistemic injustice, hidden curricula and structural reproduction of inequity. Reflexive dialogue within the team was used to discuss divergent readings of the data. Reflexive memos and team discussions were used throughout analysis to question how the researchers’ professional authority, institutional proximity and normative commitments to social justice shaped theme development. By making these dynamics visible, the study sought to enhance transparency, interpretive integrity and qualitative rigour recognising that findings represent situated interpretations rather than claims to objective or universal truth.

### 2.5 Thematic analysis

Data were analysed using reflexive thematic analysis (32) and informed by a Freirean theoretical framework. This approach was selected as it recognises the active interpretive role of the researcher in knowledge production and is suited for examining issues of power inequality and agency within educational contexts. A Freirean theoretical framework informed the analysis by drawing attention to sensitivities around issues of power and inequity with educational environments. This guided the interpretation of issues on hidden curriculum and structural inequities. However, consistent with a reflexive thematic analysis approach (32), coding and theme development remained flexible and interpretive. For example, alongside the hidden curriculum and inequities in medical education, the theme also incorporated participants’ sense of belonging reflecting meanings developed through engagement with the data rather than being predetermined by the theoretical framework.

The transcripts were read in full by the first author to achieve data familiarisation. Initial coding was conducted by the first author. Coding focused on how participants articulated awareness of social injustice, experiences of inequity in educational contexts and clinical settings and perceptions of agency and constraint. The first author generated initial themes (32) guided by Freirean concepts including critical consciousness, oppression, banking education, dialogue and praxis.

The second and third authors independently reviewed coded transcripts and generated themes, providing critical challenge and alternative readings informed by their respective disciplinary backgrounds in medicine and critical education. Theme development was iterative and collaborative with regular meetings to refine codes and themes and interrogate how theoretical commitments shaped interpretation with alternative interpretations considered, assumptions questioned and analytic decisions refined.

Themes were finalised and refined through a process of revisiting data, codes and theory rather than through frequency counting or consensus scoring. This is in line with Braun and Clarke’s (32) reflexive approach where themes were understood as interpretive constructions that captured patterned meaning across data sets.

The study did not seek saturation in a positivist sense instead conceptual depth and coherence was judged as analytically adequate, consistent with reflexive thematic analysis. Once the final thematic structure was identified, data was revisited again to ensure that no further new conceptual insights remained. Such reading of the data reinforced existing themes rather than extending or opening new theoretical directions ensuring that the dataset was adequate to support the development of coherent, meaningful and well developed themes.

Throughout the process, reflexive writing was used to document analytic decisions and consider the influence of researcher positionality. Only findings generated through this Freirean lens are reported in the Results.

### 2.6 Ethics and procedure

The study received ethical approval by the institutions’ Research Ethics Committee (REF:44425-A-Jun/2024-51143-1). Participation was entirely voluntary. When signing up for the focus groups, participants were presented with a participant information sheet and an informed consent form, after which they gave their consent and joined the focus group. Participants were informed that they could withdraw at any point without penalty. While confidentiality was ensured through anonymisation and secure data handling, full anonymity could not be guaranteed in the focus group setting. Participants were informed of this limitation and agreed to maintain the confidentiality of the discussion. At the end of the study, participants were thanked for their participation. Due to the first author’s role as an educator in the medical school, due ethical considerations were followed. The focus group study with the students was conducted by a colleague from another medical school.

## 3 Results

The data from the focus groups were analysed using Freirean framework and organised into clear thematic categories.

### 3.1 Theme 1: emerging critical consciousness -growing awareness of social justice and inequity

Scholars have argued that medical schools have an obligation to “foster clinicians who are conscious of social determinants of health, social inequality and social dynamics in healthcare” (17). This theme captured students’ early development of critical consciousness, as they identify and articulate the social, structural and institutional inequities shaping health and education (15, 29, 46).

Student participants primarily linked social justice to equitable access to healthcare, reflecting the wider literature on social justice in medical education (2, 9).

For most of them, the lack of equitable access to healthcare was a social justice issue:

*I think (social justice) means health inequities to me….anything that limits a patient’s ability to receive care so you know, it could be financial situation, it could be their age, gender and sexuality, but there’s a lot of factors that kind of play into. (FG3, P1).*

*The thing for me what came to mind first is patient receiving different type of care because of the profile that you know that they are presented with. (FG 3, P2).*

*I guess it’s (social justice) the distribution of care to the, you know, people and in fairly, uh, not just equality, but with equity as well, because there are some people that just needs it a little bit more than others. (FG 1, P3).*

Participants drew from the knowledge they gained in the classroom, identifying structural barriers that affect health outcomes such as unequal distribution of wealth, opportunities and privilege.

*I mean I think that’s something we definitely covered in class with regards to all the things that can attribute to those kind of things like, you know, uh education, you know, poverty level, those kind of things that can lead to or access to healthcare all the rest of these things I think with regards to your question about social justice, I think our understanding of its definition is like the distribution of, you know, like wealth and opportunity and privileges throughout society of with regards to it as it applies to medicine. (FG1, P3).*

*I think there tend to be, I think, uh, qualities for vulnerable people think either people with disabilities or mental health conditions. I think the I think the these are the more, umm likely populations to face inequality within the healthcare system (FG 2, P3).*

They identified income, education, geography, accessibility, poverty and housing and immigration status as structural barriers to healthcare access and outcomes.

*That’s in a way for me, it’s a little bit more in terms of accessibility and delivery of health care in terms of how that* var*ies. Again, factors like geographical and socioeconomic status. (FG 2, P2).*

These reflections speak to the wider literature on social justice, health inequities and the barriers that affect access to health and health outcomes, where social justice is defined as addressing and removing health inequities (47). Students showed a good understanding of the social determinants of health, recognising the barriers that impact health access and outcomes, which include education, poverty, disabilities etc (8, 9, 14).

One student recognised that racism, sexism and other systemic inequities might affect health outcomes.

*To me, social justice is identifying, perhaps like EDI related barriers such as, you know, racism or classism or issues and working proactively to make sure that those issues do not affect people that within those groups that are typically affected. (FG 3, P4).*

This reflection by the student participant is often an under-stated but critical component of health inequities. Scholars have identified how discrimination including racism play a role in exacerbating health inequities (34, 35). Covid-19 pandemic highlighted how racism in healthcare was a significant driving force behind the disproportionately high mortality rates in minoritised groups (34). Sim et al. (35) in their systematic review of the perspectives of health professionals and patients on racism in healthcare state that most countries do not directly address racism towards patients. They call for addressing these issues early in the curriculum and the reflection above confirms that these issues were incorporated in the curriculum.

Participants also identified structural barriers within their curriculum that affects patient outcomes.

*I think the medical profession is rampant with them like uh, medical diagrams tend to be white males. Female populations tend to be underrepresented as far as, like scientific research studies, and I know there’s like a big movement right now to try to combat those issues, but the underlying focus of the education and a lot of the science we have been relying for relying on has been skewed over time. So we are always dealing with that at the core, I think (FG 3, P4).*

This student participant recognised how the curriculum contributes to health inequities. They reflected on how educational materials including diagrams focused on white males and knowledge based on research conducted on males affect their ability to treat patients from diverse groups (2020; (5, 10, 11)). The underrepresentation of minoritised bodies in medical curricula has direct implications for diagnostic accuracy and patient safety. Studies demonstrate that the predominance of white, male bodies in teaching materials contributes to diagnostic errors, particularly in conditions where clinical presentation varies by skin tone, sex or ethnicity (11). This epistemic bias not only undermines clinical competence but reinforces structural racism within healthcare systems by privileging certain bodies as the normative standard (5).

This theme reflects students’ initial development of critical consciousness, as they demonstrate a strong conceptual grasp of social justice, health inequities and the social determinants of health (9). Freire’s concept of critical consciousness; the awareness and questioning of power and privilege is conceptualised in medical education as the focus on an understanding of the social, cultural and historical dynamics that affect health outcomes (17). The reflections in this theme evidence that students recognise how racism, socioeconomic conditions and the medical curriculum shape patient access and outcomes. This awareness reflects the early stages of Freire’s conscientization, an awakening to the political and ethical dimension of healthcare work (8, 17).

### 3.2 Theme 2: witnessing inequities in clinical practice

Here, the students reflected on their encounter with real-world manifestations of systemic injustice, drawing from their experiences in GP placements. Their reflections were consistent with Freire’s idea that oppression becomes visible when learners engage with lived experiences.

In their GP placements, students were able to witness patient experiences which they were able to link to structural inequalities including, neurodivergent patients not being fully supported or understood by providers and patients facing language barriers.

*I think from the patient's end, there have been many inequalities… sometimes patients come in and one of the patients that I worked with personally…patient that was neurodivergent that didn’t have the ability to kind of speak… So that was a bit of a challenge to address and just be able to work with that and understand that I may not have the appropriate, you know, training so far, even as a medical student, to be totally ok for this particular patient (FG 3, P1).*

*So yeah, I was observing a consultation where the patient did not know English and the doctor was really on assumption basis on wondering whether they understood what they were saying or not. It I think it's very important for doctors… or GPs to realize that the more you assume care to these patients, the more outcome gets worse. (FG3, P3).*

Student participants drew from their clinical placements in GP settings to highlight examples of structural determinants in practice. They gave multiple examples including transportation issues, disability related challenges, language barriers and lack of interpreters.

*…I think something I noticed through the GP placements is that the access to care is a big part in terms of it…whether that be lack of transportation or any kind of disability that and makes it hard for them to actually attend the clinic because they don’t have that point of access, and if they're not actually able to get the treatment that they need or when they need it, then that obviously you know they might miss major diagnosis. They might miss the care that they could have gotten when they actually needed it, and then that can lead to deterioration or just an overall worse outcome than if they had the access and when it's necessary because of their inequality….if they don't have the resources that other people would have. (FG 2, P1).*

They spoke about the challenges asylum seekers face in relation to lack of documentation, facing housing instability and limited resources.

*My GP placement was near an area that was a very focused with the asylum seekers and we discussed in at length the difficulties of treating patients they may not have anything beyond the passport and the backpack and be living out of a hotel room (FG 3,P4).*

Most students were also able to identify the structural constraints within GP settings which may hinder the care of patients. They observed that these settings often face insufficient resources in relation to interpretation services or helping disabled patients with transport

*What I saw was a parent and child walk in and the doctor asked question like who’s the patient? And she asked in… English. And both of them were looking at each other and like all you know, and she assumed the parent was the patient. So she was trying to talk to the parent and only the child knew English. So it was a really confusing consultation where she’s trying to talk to the child who’s basically only six years old or something, trying to get them to talk to the parent in their home language. And it was kind of where I felt like that’s when we should really include it in the GP's as well, like I know they have interpreters, why aren't they getting used in real life? Like, why aren't they getting used right now? (FG3, P3).*

*So in this in this instance, this patient was unable to get to the clinic because she was disabled and her child was also disabled. So she had no access to the clinic. So I was one thing I think I brought up was if it's possible to pick up the patients from their homes and then take them to the clinic. But then the GPs is also explaining to the patient herself that like they just don't have the resources to do it, they need a driver for that. They need a van for that, and it's just unfeasible. So I think when I did kind of bring it up, it was more of like a ohh like the resources not available and there's only so much they can do. Obviously, they can make other adjustments like change the dates or, umm, arrange things that are within their control, but you can't do everything you know. Which is fair and I feel because always that clinic was also very big. They had a lot of patients, so it made sense, so I didn't know what I could do really (FG 2, P1).*

Participants recognised that staff were aware of the challenges but had no organisational capacity to respond

*Well, luckily, we were the only one who knew the language. Me and my friend were observing the consultation so we figured we were not allowed to speak… when I don’t know how comfortable with that patient would be to talk to a stranger about her problems and then wanting me to translate it to the so, I think that’s where the barriers come in as well. So even though you want to help, there’s only so little you can do when it comes to real life. Like when it's happening in front of you, there’s only so little you can do. Rather than be like, I suggest that you have a translator in this GP or like I suggest you should….you can only put in the suggestion box and leave (FG 3,P3).*

The above reflections illustrate that students were able to connect individual patient experiences to wider structural issues in clinical environments including lack of capacity, time and resources. The participants’ experiences reflect the structural challenges recognised in the literature. Empirical studies highlight that structural barriers within healthcare systems significantly shape patient experiences and outcomes, particularly for marginalised groups. For example, research demonstrates that limited access to professional interpreters contributes to miscommunication, diagnostic delays and reduced patient safety, disproportionately affecting patients with limited English proficiency (36). Accessibility barriers, including inadequate transport provision, inaccessible clinic environments and insufficient accommodations for disabled patients, further compound inequities in care (14). Resource constraints within primary care such as time pressures, understaffing and limited availability of social support services have been shown to restrict clinicians’ ability to address social determinants of health, reinforcing systemic disadvantage (46).

Students linked structural inequities to outcomes as they identified recovery rates, mortality, missed diagnoses due to poor access and reduced quality of care in underserved areas

*And if they’re not actually able to get the treatment that they need or when they need it, then that obviously you know they might miss major diagnosis. They might miss the care that they could have gotten when they actually needed it, and then that can lead to deterioration or just an overall worse outcome than if they had the access and when it's necessary because of their inequality. (FG 2, P).*

The reflections in this theme highlight students’ recognition of how oppression operates systemically and the constraints within clinical practice to support these patients to overcome structural barriers. Sharma et al. (37) state that simply teaching about health inequalities and structural barriers are not enough, they argue that teachers need to show the students “conditions to be challenged and changed.” (17). The reflections of the student participants highlight Freire’s assertation that oppression becomes visible when individuals critically examine real world conditions rather than understanding theoretical concepts. Student observations in this theme demonstrate an awareness that inequity is systemic rather than isolated and that clinical structures; time pressure, resource scarcity and hierarchical protocols often reproduce disadvantage (38). The accounts of the students in this theme highlight how they “learn with and in the world” as prescribed by Freire (39).

### 3.3 Theme 3: the hidden curriculum - inequities and belonging within medical education

This theme reflects Freire’s assertation that educational institutions reproduce social hierarchies through implicit norms and structures. Manca et al. (17) in their scoping review of critical consciousness in medical education identify hegemonic discourses within an increasingly international and globalised medical education.

During the focus group discussions on social justice, students highlighted financial inequities, where financial privileges enabled them to access medical education in the UK.

*XX (country) is impossibly hard to get into medical school… they don’t have enough spaces for the amount of people that want to become doctors…You can't get into medical school unless you have more than eight stars in everything and really perfect SAT’s. People, therefore, going outside the country to study medicine, if you don’t have the money to do it, you can't become a doctor. And then this career is literally closed for you….(FG1, P2).*

Students also recognised differing access to private tutoring or learning resources and how this affected their performance

*Like in terms of like the financial background that each student say, some students are able to get access to more learning materials online because they’re able to pay for it. Some not, because, you know, they’re probably on the on the more of a tighter budget monthly. Some can get tutors, some not like these. You can see that everywhere. (FG3, P2).*

Together, these represent examples of the hidden curriculum of inequality, where students learn that medical education mirrors broader social injustice. Entry to medicine is not meritocratic, a number of social inequalities impact affect their chances to gain entry (40). Their progress in medical school is not entirely meritocratic as pointed out by these students (41); financial privilege enables them to gain advantages through extra tutoring. Financial disparities shape students’ access to educational resources, with evidence showing that students from lower socioeconomic backgrounds face barriers to purchasing supplementary materials, private tutoring or exam preparation resources, contributing to differential attainment (24). Such financial inequalities may have been in the forefront of their reflections given their international student status.

Evidence from the literature demonstrate that students from minoritised backgrounds encounter microaggressions and discrimination, which negatively impact their sense of belonging, engagement and academic attainment within medical education learning environments (5, 18, 24), the students in this study did not report.

However, most student participants in the study revealed that they felt a sense of belonging in the medical school and identified factors that contributed to creating a sense of community and belonging.

*I think there’s a really strong sense of community for me… it’s quite a small group of and we’re all international students too…felt like there was some sort of like camaraderie from the start….and we had obviously our TBL groups, I think that's probably a big thing that comes with the sense of belonging because we were initially we all just checked into a group of seven and you're with them for the whole year….you have a small little group of people that, you know pretty well, you get to know really, really well over the first year… I think that definitely eases that sense of belonging and that sense of community coming into the medical school*. (FG1, P4).

The participant above echoed the reflections by several students that peer relationships, supported by the Team-based learning structure fostered belonging and created a sense of continuity. This shows that stable peer teams enhance belonging, reduce isolation and support academic transitions to new environments, this is especially important to international students and minoritised learners.

Most students also reflected on the role played by staff to shaping belonging, stating that they were approachable and caring.

*When I first got to XX it was the first time I was in the UK, so everything was new and I never been here. I didn’t have friends. Yeah, and it was really scary for me and I think. Umm, one thing that I’ve that really stayed with me was how the staff they were really, really welcoming and it didn't really feel like it was like staff and students, they felt like they were also just there for you. And like there for you, if you wanted them, if you wanted help or any kind of help or help at all or any kind of talk. They were open and willing to listen, and I think, as in an international cohort…(FG2, P1).*

The above reflection speaks to the evidence in the literature that belonging is fostered when staff are approachable, empathetic and invest in student wellbeing.

Participants also reflected on the institutional efforts to support international learners. Participants described policies and support services that made them feel recognised and valued.

*I just wanted to say that… I think I definitely belong to the medical school in terms of I think going back to the policies, I think they do have many things in place to kind of especially for international students to like to feel that they belong or that they would feel comfortable….I feel like there is a lot of things put in place, either by the support team or by the program team to make sure that everyone feels included and then they're not missing out (FG2,P3).*

This shows that belonging can be created by intentional institutional design. This speaks to the evidence in the literature that belonging is not just felt but structurally designed, shaped by the curriculum, policies, learning design and institutional strategies towards equity (24, 25).

The sense of belonging described by participants aligns closely with Freire’s principles of dialogue, humanisation, and relational justice. Feeling welcomed by staff and embedded within stable, supportive peer groups created the psychological safety necessary for students to engage critically with social justice issues. For international students in particular, belonging can counteract the challenges associated with studying in a new country, enabling the kind of relational trust and community that Freire viewed as foundational for developing critical consciousness. In this way, belonging can be understood not simply as an affective experience, but as a Freirean condition for transformative, justice-oriented learning. At the same time, this sense of belonging highlights a tension within the wider findings: while relational inclusion is fostered locally, broader structures continue to limit students’ agency to enact the justice-oriented values they are taught.

### 3.4 Theme 4: banking education and students as agents of change

This theme reflects Freire’s critique of the banking model where learners absorb information but lack real power to enact change.

Participants spoke about their awareness of inequalities but did not believe they were expected to address inequalities. They felt that they were not equipped or empowered to act. They felt that meaningful action was only possible once they graduate.

Some students shared their feeling of limited agency to enact change.

*I feel that the few would get a chance. Obviously, yes. We would like to contribute and going in the field, obviously you want to make change because tomorrow you will also be treated by some Doctor who would probably have learned something about along these lines and probably wants to make a change as well. So yes, 100% if you get a chance, yes. (FG 3,P3).*

*I think at this point I don't think we do have the position to maybe address some of the inequalities, but I think we, but we do have the I think that are able to maybe you're reflect on it and maybe if there's something that's worth reporting to this good. And I think there is everything for that, but maybe I think more our at FY1 and FY2 would have the wider opportunity to maybe address some of the any inequalities and maybe have some of them. (FG 2, P3).*

Most student participants below reflected that at this stage of their programme their education was primarily about awareness and knowledge rather than address or act to change conditions.

*Not sure if that's the thing that they should be focusing on just allowing identifying that, hey, these are problems that need to be faced and us as incoming medical professionals, we need to be mindful of those kind of things and then keep our keep our eyes out for possible solutions (FG 1, P3).*

*I think I think it's more to do with the awareness of this at this point in time, because I mean, we're really entering year three but I think we on the whole we would feel relatively open to tell our clinical supervisor about something major. So I think at least having that awareness at this point in time, that’s most important (FG 1, P2).*

*I think the education that we're provided with now is more of an awareness type of education. Like, I don't think they're necessarily trying to be like, hey, this is your responsibility. I think they do tend to remind us sometime that we do as incoming medical professionals that we are going to play a role in it. (FG 1, P3).*

They identified that their ability to address inequalities were constrained by lack of resources and policy constraints.

*I feel like what you've just said is like we've is like one of the reasons we've all probably got into medicine like to obviously help individuals….So obviously we can try be that change. However, it's very difficult when you've got a whole system say that the NHS or the government isn't willing to fund, and obviously that's going on now with all the strikes going with junior doctors and also consultants. So it's very difficult like learning about it and then wanting to be that change. But obviously coming to then implementing that when we graduate will be difficult (FG 1, P1).*

Some students recognised the power of collective action and spoke about joining advocacy groups.

*I think I think now maybe now ways to do that at least would be like joining different organizations, for example like BMA that does advocacy etcetera for you know equal access, safe access to healthcare surgery. Eventually I think it depending on what, what pathway you go down really whether or not you’re passionate about these things. But I do see like there are there are things to be changed and I know people around me that are getting involved. (FG 3,P1).*

*I think I’ve especially realized that within Medicine as an institution, it’s a it’s a numbers game. I don’t think the individual has as much power as maybe it’s perceived, but getting groups together that have a particular focus seems to be a more effective Avenue through my experience at Brunel, as far as making medical change. (FG 3, P4).*

This mirror’s Freire’s banking model, where students receive knowledge but are not empowered to critically transform their environment. Theme 1 and 2 revealed that the student participants displayed emerging critical consciousness as they embodied reflexive awareness of the issues, however, the reflections in this theme (4) revealed that they did not identify with the role of medical students to foster transformation (17). The participants reflections emphasise feelings of powerlessness, uncertainty about their role to enact change and lack of structural opportunities to bring about change. This echo O’Brien et al. (46) argument that while medical education increasingly promotes awareness of social justice issues, it often fails to provide opportunities for students to engage in meaningful action. The literature identifies the critical role of medical educators to foster action-oriented attitudes in the education of healthcare professionals (42, 43) and calls for medical schools to be socially accountable and lead change (8, 17). Scholars have called for curricular that cultivate students’ potential as change agents (44, 45) and participate actively and meaningfully in society (17).

## 4 Discussion

This study explored how international medical students conceptualised social justice, how they experienced inequity within clinical and educational environments and how they perceived their capacity to act as agents of change. Using a Freirean analytical frame provided a powerful lens to understand the complex relationship between students’ developing awareness of injustice and the structural conditions that limited their emerging sense of agency. The findings highlight a clear tension between awareness and agency, and between aspirational commitments to social justice and the structural realities of hierarchical and resource pressured systems within which students learn.

### 4.1 Students’ conceptualisation of social justice: awareness without agency

A central finding in the study is that students possess a strong conceptual awareness of social justice, health inequities and the social determinants of health. This is in line with the predominant literature in medical education where social justice is framed around fairness in the allocation of healthcare resources and the reduction of health disparities (4, 6, 7, 47). Participants’ reflections show early stages of critical consciousness recognising how racism, poverty, disability, immigration status and language barriers as drivers of unequal outcomes (14).

A strong analytic outcome of the student was the emerging sense of critical consciousness amongst the student participants indicated as expressions of intent to act, resistance to dominant norms, recognition of structural vs. individual responsibility.

Through a Freirean theory lens, this suggest that while students embodied an emerging sense of critical consciousness, in relation to the transformative agency integral to critical consciousness (1, 4, 17), the curriculum functions predominantly as “banking education” where learners are provided with information about injustice but not supported to apply this knowledge in transformative ways. Students’ reflections on “not having power yet” “not being able to intervene” or being “only observers” demonstrate how the system encourages reflection but restricts actions. This disconnect between critical awareness and structural agency represent a key barrier to enacting social justice in medical education. Students are taught about inequity, provided opportunities to observe these in practice but remain structurally unable to intervene in meaningful ways. This suggest that while medical curricular succeed in fostering awareness, developing empowered justice oriented practitioners remains limited.

### 4.2 The hidden curriculum and belonging

The study also revealed that the influence of the hidden curriculum in shaping students’ experiences. While formal teaching promotes values of equity, inclusion and patient-centred care, students encountered systemic inequity; over stretched GP services, limited interpreters, inaccessible services for disabled patients and challenges faced by asylum seekers. Students perceived these inequities as systemic rather than individual failures or issues. Their position within the clinical hierarchy meant that while they could recognise injustice, they could not change it or challenge it. This reinforces previous research which show that hierarchical norms and time-pressured environments inhibit learners willingness and ability to address concerns or propose alternatives (15, 16).

Students identified aspects of the hidden curriculum within educational environments, particularly financial inequities that influence learning opportunities and success. They also recognised representational inequities in curricular materials (5, 11, 24). However, other structural inequities in medical education well documented in the literature; such as attainment gaps, discrimination and experiences of marginalisation affecting belonging (21–24) did not feature in the reflections of the students.

Instead, students identified a strong sense of belonging reflecting a relational dimension of social justice, in which students feel recognised, valued, and supported within their educational environment. These findings suggest that belonging is not merely an affective experience but a justice-related one; it creates the psychological safety necessary for learners to question power, engage in dialogue, and participate in the early formation of critical consciousness. Freire argues that dialogue, critical reflection, and transformation can only emerge in environments where learners experience dignity and relational safety. Participants described meaningful peer relationships, stability within Team-Based Learning groups, and supportive staff who were approachable, welcoming, and emotionally available. For many, particularly those entering the UK for the first time, this sense of community mitigated early isolation and vulnerability. For international students navigating unfamiliar cultural and educational systems, belonging also functioned as a protective factor against marginalisation, aligning with Freire’s emphasis on understanding learners within their social and political contexts. Their reflections demonstrate that belonging is not a peripheral emotional experience but a relational foundation for developing critical consciousness.

From a Freirean perspective, the presence of belonging also sharpened the central contradiction illuminated by this study: although medical schools may foster inclusive communities, structural and institutional barriers continue to limit students’ capacity for transformative action. While students felt supported by their immediate community, they still perceived broader systems; clinical hierarchies, resource-limited environments, bias in curriculum, as reinforcing inequity and restricting agency. This tension highlights that relational inclusion does not necessarily translate into structural empowerment.

### 4.3 The international student context

This study makes a distinctive contribution by focusing on international students’ perspectives, an underrepresented group in social justice and HPE scholarship. For these students experiences of inequity are shaped by their identities, complex positionalities within UK medical education, navigating financial burden, cultural adaptation, and varying degrees of belonging within institutional hierarchies (21, 24). Their reflection on financial inequities in accessing medical education reveal a heightened sensitivity to the socioeconomic factors shaping entry into the profession. Furthermore, study highlighted how international students develop critical awareness of structural inequities unique to the UK context, through early exposure to clinical environments. Many enter UK programmes with prior educational and sociopolitical experiences that inform and shape their understanding of justice. While these dynamics can create feelings of marginalisation or limit opportunities to participate in advocacy, their reflections in the study did not convey experiences of marginalisation within educational environments. They did however, convey a strong sense of belonging, which played a crucial role in shaping how they navigated the early stages of medical training. Their accounts of supportive peer networks, stable learning groups, and approachable staff suggest that the programme fostered what Freire describes as humanising educational relationships, in which learners feel recognised, respected, and valued. This relational ethos created conditions for authentic dialogue, a core Freirean principle, enabling students to explore complex questions about inequity from a position of psychological safety rather than fear or alienation. For international learners who may otherwise experience cultural displacement, belonging functioned as a protective factor that facilitated engagement with justice-oriented learning. In this sense, belonging becomes a form of relational justice, affirming students’ dignity and supporting the early formation of critical consciousness.

### 4.4 Towards transformative justice in medical education

The findings highlight the need for medical education to move beyond awareness raising and towards transformative approaches that develop meaningful opportunities for praxis. While curricular address social determinants of health there remains limited structural support for students to engage in action and advocacy. Creating such opportunities is critical for developing practitioners capable of advancing equity within and beyond education to clinical practice.

Implication for curriculum design and faculty development.

1. Embed formal structured opportunities for action-oriented learning, such as community engagement projects, advocacy pathways and involvement in quality improvement initiatives early on the curriculum.
2. Retain stable, small-group learning structures (e.g., TBL, learning communities).
3. Incorporate early induction programmes tailored to international learners.
4. Embed cross-cultural peer mentoring.
5. Build intentional opportunities for relationship-building across the programme.
6. Provide opportunities for students to co-construct knowledge, interrogate power and practice speaking up.
7. Critically review curricular content to address epistemic and representational injustices.
8. Support educators to recognise power dynamics and equity focused practice.
9. Develop skills to facilitate discussions about injustice with diverse and international cohorts of students.

### 4.5 Limitations and future research

This study draws on a small sample of participants from a single institution and focuses on Year 2 students, whose experiences may differ from a later stage of training. Future research could explore longitudinal development of critical consciousness and compare the trajectories between UK home students and international students or examine faculty conceptualisation of their own roles in supporting social justice in medical education.

## 5 Conclusion

This study examined how international medical students conceptualised social justice, how they encounter inequity within educational and clinical environments, and how they perceive their capacity to act as agents of change. Through a Freirean lens, the findings illuminate a clear tension between what students are taught about justice and what they are structurally empowered to do. While participants demonstrated strong early forms of critical consciousness; recognising how poverty, racism, disability, immigration status and biased curricular structures shape patient care, they also perceived barriers that limited their ability to translate this awareness into meaningful action. For international students in particular, this awareness of complex structural factors affecting health equity was supported by their early exposure to clinical environments in GP placements.

Although students reported limited agency in addressing systemic inequities, they simultaneously described a strong sense of belonging within the medical school, fostered by stable TBL groups, supportive peer communities, and welcoming staff. These experiences of belonging represent an important relational dimension of social justice. In Freirean terms, belonging reflects humanising educational relationships in which learners feel recognised, valued, and safe to participate in dialogue. This relational inclusion provided international students with a foundation for navigating unfamiliar cultural and educational environments and helped create the psychological safety needed for early engagement with critical reflection.

However, belonging alone could not overcome the broader structural constraints students faced. Resource limitations in clinical settings, elements of the hidden curriculum, and entrenched hierarchical norms continued to restrict their opportunities for justice-oriented action. This contrast underscores a central contradiction in medical education: while students may experience supportive communities that affirm their identity and belonging, the wider structures within which they learn still reproduce the inequities they are expected to challenge. Thus, even within relationally inclusive environments, learners remain limited in their ability to move from awareness to praxis, highlighting the systemic nature of the agency gap.

A transformative approach to social justice education is therefore needed, one that moves beyond teaching about inequity toward developing the skills, confidence, and institutional conditions required for students to act upon it. This includes creating structured opportunities for praxis, fostering dialogic and participatory pedagogies, and explicitly addressing the hidden curriculum. Such approaches should also recognise the importance of belonging as a relational foundation for justice-oriented learning, while ensuring that belonging is accompanied by genuine opportunities for engagement, influence, and structural participation. Faculty and institutions must work intentionally to support students, especially international learners, in developing agency within systems that often constrain their voice.

By foregrounding the perspectives of international medical students, this study contributes new insights into how social justice is understood within UK medical education. It highlights the need for curricular and institutional cultures that not only cultivate critical awareness and belonging but also actively support students in transforming unjust structures. Supporting learners to integrate reflection with action is essential for developing critically conscious, socially responsive, and justice-oriented future practitioners.

## Data Availability

The original contributions presented in the study are included in the article/supplementary material, further inquiries can be directed to the corresponding author.
